# Supplementary material for: Identification of photoperiod-induced specific miRNAs in the adrenal glands of Sunite sheep (Ovis aries)
Source: Front Vet Sci. 2022 Jul 22;9:888207. doi: 10.3389/fvets.2022.888207 (PMC9354845; doi:10.3389/fvets.2022.888207)
Supplement: Supplementary file 1 [file Data_Sheet_1.ZIP › Supplementary file/miRDeep2 file.zip!/miRDeep2 file/Know_Structure/LP42A2/oar-mir-665.pdf]

miRBase precursor : oar-mir-665
Total read count : 91
oar-miR-665-5p read count : 3
oar-miR-665-3p read count : 85
remaining reads : 3

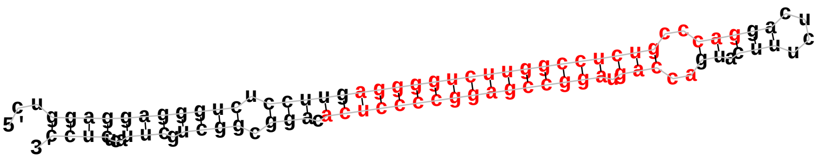

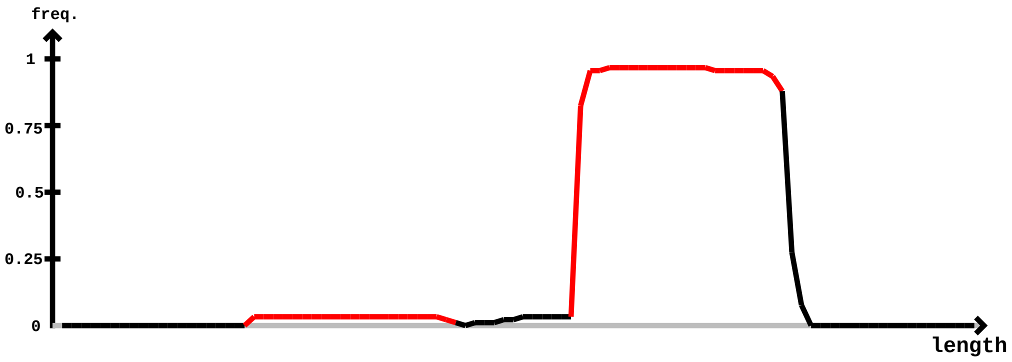


```
                                                      oar-miR-665-3p
                        oar-miR-665-5p
5'- cuggaggagggucuccuugaggggucuuggccucugcccaggacucuuucaugaccaguaggccgaggccccucacaggcggcugcuuacucucc   -3'    exp
    ..((((((((((.(((((((((((((((((((((((..(((((....))).))..))).))))))))))))))))).))).)))).))).))))..   reads      mm        sample
    ...................aggggucuuggccucugccc........................................................   1          0         seq
    ...................aggggucuuggccucugccca.......................................................   1          0         seq
    ...................aggggucuuggccucugcccag......................................................   1          0         seq
    ..........................................acucuuucaugaccaguaggccgaggccccu......................   1          0         seq
    .............................................cuuucaugaccaguaggccgaggccccuca....................   1          0         seq
    ...............................................uucaugaccaguaggccgag............................   1          0         seq
    .....................................................accaguaggccgaggccccu......................   1          0         seq
    .....................................................accaguaggccgaggccccuc.....................   5          0         seq
    .....................................................accaguaggccgaggccccuca....................   52         0         seq
    .....................................................accaguaggccgaggccccucac...................   11         0         seq
    .....................................................accaguaggccgaggccccucaca..................   3          0         seq
    ......................................................ccaguaggccgaggccccuca....................   2          0         seq
    ......................................................ccaguaggccgaggccccucac...................   7          0         seq
    ......................................................ccaguaggccgaggccccucaca..................   3          0         seq
    ........................................................aguaggccgaggccccucaca..................   1          0         seq
```
